# Male gonadal axis assessment in bariatric surgery candidates

**DOI:** 10.1186/1758-5996-7-S1-A136

**Published:** 2015-11-11

**Authors:** Gabriela Ghisi e Ghisi, Giovana De Nardin, Fernanda Augustini Rigon, Maiara Ferreira Peixer, Marisa Helena César Coral, Simone van de Sande-Lee, Marcelo Fernando Ronsoni, Alexandre Hohl

**Affiliations:** 1 H.U- Hospital Universitario de Florianópolis, Florianópolis, Brazil

## Background

Obesity is increasingly prevalent worldwide and has profound impacts on health and quality of life. It is known that testosterone plays an important role in the pathology of metabolic diseases such as obesity. Although very prevalent, this association is underdiagnosed.

## Objective

To evaluate the male gonadal axis in patients with body mass index (BMI) ≥ 35 kg/m2.

## Materials and methods

Cross-sectional study, including male patients evaluated for bariatric surgery that had a BMI ≥ 35 kg/m2. Blood samples were collected in the morning, after overnight fasting, and all tests were performed in the same laboratory.

## Results

We evaluated 69 subjects, mean age 39±10 yrs. and 87% caucasian. Type 2 Diabetes Mellitus (T2D) was found in 47.8%, hypertension in 72.5%, dyslipidemia in 23.2% and metabolic syndrome according to the IDF in 87%. Mean weight, waist circumference and BMI were respectively: 157.4±31.0 kg, 148.2±14.9 cm and 51.2±8.3 kg/m2. The average fasting glycemia was 111.1±34.7 mg/dL (NR <100), HbA1c 6.5±1.3% (NR <5.7), total testosterone (TT) 232.8±96.9 ng/dL (NR >300), LH 3.1±1.3 mUmL (NR 0.8 to 7.6) and calculated free testosterone (CFT) 5.9±2.7 ng/dL (NR> 6.5). 79.7% of subjects had TT ≤ 300 ng/dL and 56.5% CFT ≤ 6.5 ng/dL. Categorizing patients according to the levels of TT [G1 (≤ 200 ng/dL): 53.8±8.5 kg/m2 x G2 (201-299 ng/dL): 49.3±7.2 kg/m2 x G3 (≥ 300 ng/dL): 48.7±8.7 kg/m2], there was a statistically significant difference only in relation to BMI (p=0.04). There were no statistically significant differences in mean TT and CFT between individuals with and without T2D [TT: 218.8±89.4 x 245.5±102.8 ng/dL (p=0.25); CFT: 5.65±2.7 x 2.6±6.21 ng/dL (p=0.39)].

## Discussion/conclusion

The evaluation of our group of patients with BMI ≥ 35 kg/m2 showed a high rate of individuals with TT less than 300 ng/dL. We also identified a statistically significant difference in BMI according to the categorizations of TT, with higher BMI levels in patients with TT ≤ 200 mg/dL. In this sense, it reinforces the need for gonadal axis assessment in obese patients and their appropriate monitoring and treatment.

